# Investigating the association between night eating symptoms and chronotype: the mediating role of depressive symptoms in a sample of Italian university students

**DOI:** 10.1007/s40519-024-01707-y

**Published:** 2025-03-15

**Authors:** Giulia Riccobono, Tommaso Barlattani, Valentina Socci, Edoardo Trebbi, Angela Iannitelli, Assunta Pompili, Francesca Pacitti

**Affiliations:** 1 Department of Biotechnological and Applied Clinical Sciences, University of L’Aquila, Via Vetoio, 67100 L’Aquila, Italy; 2 Department of Public Health and Infectious Diseases, “La Sapienza” University of Rome, 00100 Rome, Italy

**Keywords:** Night eating syndrome, NES, Night eating symptoms, Chronotype, Depression, Eveningness

## Abstract

**Purpose:**

This study aimed to understand the relationship between night eating symptoms, chronotype, and depressive symptoms among Italian university students.

**Methods:**

The study assessed 905 students using self-report questionnaires, including the night eating questionnaire (NEQ), the Morningness–Eveningness Questionnaire (MEQ), and the Beck depression Inventory (BDI). The correlation between variables was analyzed using Pearson correlation analysis, and mediation analysis was conducted using SPSS PROCESS Macro to estimate the association between variables.

**Results:**

Among the students' sample, the mean age was 25.54 years, with an age range between 18 and 35, 68.7% were women, 15% were morning types with MEQ scores of 59 and above, 64.8% were intermediate types with MEQ scores between 42 and 58, 20.3% were evening types with MEQ scores of 41 and below, and 3.6% reached the criteria for night eating syndrome (NES). There was an inverse correlation between MEQ and BDI scores, higher BDI and lower MEQ scores, and a significant inverse correlation between NEQ and MEQ scores, higher NEQ and lower MEQ scores. Individuals with higher NEQ scores had higher BDI scores, indicating a significant positive correlation between night eating symptoms and depressive symptoms. MEQ had a statistically significant negative direct effect on BDI and NEQ variables. The direct impact of BDI on NEQ was positive and statistically significant. The indirect negative effect of MEQ on NEQ through BDI was also determined to be statistically significant.

**Conclusion:**

The study found that depressive symptoms played a significant mediating role in the link between eveningness and night eating, with a partial mediation. Evening chronotype was associated with an elevated night eating score. The findings emphasize the importance of chronotherapeutic approaches in treating night eating. However, further research is necessary to elucidate the intricate relationship between these variables.

**Level of evidence:**

Level III. Evidence obtained from well-designed cohort or case–control analytic studies.

## Introduction

Circadian rhythms significantly impact our sleep–wake cycle, wakefulness, and peak performance times [1]. Chronotypes refer to an individual's preference for the timing of their sleep–wake cycle and are part of their circadian rhythmicity [2]. They can be categorized into three types—morning, intermediate, and evening—reflecting an individual's activity and sleep patterns from early morning to late afternoon [3].

People with a morning chronotype tend to have an earlier sleep–wake cycle, and their mental and physical performance peaks in the morning [4]. On the other hand, those with an evening chronotype prefer to go to bed later and wake up later in the day [5]. Chronotypes are influenced by age, with a shift towards being an evening person in early adolescence and a constant transition to becoming a morning person as one ages [6]. Adolescents tend to have a delayed sleep and activity pattern, so it may be expected that a more significant number of young adults will report evening-type preferences than morning-type preferences [7]. In a study on a large 2135 Spanish university student sample, 16% were classified as morning, 60% as intermediate and 24% as evening types [8]. Riccobono et al. [9] in research regarding 1136 students at the L’Aquila University, Italy, reported a percentage of around 15.3% for Morning Type, 64.3% for Intermediate and 20.4% for Evening Type.

Recent studies have identified a strong link between an individual's chronotype and their mental health. The relationship between chronotype and emotional regulation processes has recently been investigated [1]. Watts and Norbury's study [10], found that expressive suppression was positively associated with eveningness, while morningness was positively associated with cognitive reappraisal strategies. Specifically, the evening chronotype has been associated with an increased risk of depressive disorders, while the morning chronotype is considered protective against such disorders [2]. Longitudinal studies have also shown that depression in adolescents can lead to a greater preference for staying up late, and the eveningness dimension has been linked to the later development of depression, highlighting a robust bidirectional relationship [11].

Individuals with an evening chronotype are more likely to experience circadian disturbances and changes in their eating behaviors [12]. This can affect their circadian preference, making them more prone to eating disorders than healthy individuals [13]. The food intake for humans occurs during the active phase, allowing for the replenishment of energetic reserves [14]. Conversely, the depletion of energy stores and the fasting period occurs during the non-active phase, which includes sleep [15]. This cycle is one of the strongest zeitgebers, in synchronizing circadian rhythms in peripheral tissues [16]. Thus, it may be one of the main issues in individuals with a disturbed circadian pattern, particularly in subjects that could suffer from night eating, like night-time workers [17].

Studies have shown that there is a bidirectional relationship between chronotypes and eating disorders, with changes in eating behaviors impacting an individual's circadian preference [18]. Night eating behavior has been closely linked to a delayed sleep phase and directly affects differences in chronotype [19]. Although the existing literature does not agree on the relationship between night eating behaviors and chronotypes, robust findings suggest a significant association between night eating behaviors and evening chronotypes [9, 20–22]. Additionally, night eating behavior has been associated with depressive symptoms in women [23] and young adults [24]. Interestingly, an association was found between levels of depression and episodes of night eating [25]. Night eating [26, 27], along with evening hyperphagia (consuming more than 25% of total calorie intake after the evening meal) [28] and loss of appetite in the morning are the core symptoms of Night Eating Syndrome (NES). This disorder has been recently recognized as a distinct disease in DSM-5 and is categorized under 'Other Specified Feeding or Eating Disorder' [29]. The prevalence of NES is 1.5% in the general population of the United States [30] and 8.2% among university students [31]. Individuals diagnosed with depression or depressive symptoms, particularly students [31], are at a significantly higher risk of experiencing NES [32, 33]. Several studies [34–36] have suggested a link between NES and depressive symptoms. Moreover, more than 50% of NES patients were found to be comorbid with major depressive disorder throughout their lifetime [37]. Indeed, NES frequently exhibit depressive symptoms that worsen in the evening hours [34–36]. Moreover, NES has also been associated with higher body mass index (BMI); however, literature concerning the association between night eating and BMI produced mixed findings, with emotional eating and age as potential moderators of this relationship [38].

Different studies [9, 19, 22] have pointed out that individuals with NES score lower on the MEQ, indicating an inclination towards an evening chronotype. Conversely, one recent study has proposed an association between the morningness dimension and NES [39] in a population of Greek non-clinical adults. Some studies [28, 34, 40] have also hypothesized that NES subjects present a circadian delay exclusively in their food intake, with unaltered circadian rhythms in global functioning. Various studies also identified a close relationship within the clinical population between the eveningness dimension and both NES and binge eating disorder [41], as well as between NES and both evening type and bipolar disorder [42–44]. A study has also established a correlation between evening chronotype, NES, and depressive symptoms in young adults [22]. Moreover, the efficacy of Bright Light Therapy (BLT) in treating night eating behavior and improving mood and sleep quality suggests the need for further investigations into the relationship between NES, mood, and circadian rhythms [45, 46]. Chronotype-related differences in eating patterns have been studied, but research on the connection between chronotypes and specific eating disorders is limited [47]. The evening chronotype was associated with food addiction in a cross-sectional survey of university students [48]. Interestingly, NES was found to be associated with food addiction more strongly in an adult community sample [49].

A large-scale study in Finland [50] investigated the relationship between chronotype, depressive symptoms, and emotional eating behavior, confirming a negative correlation between morning preference and both depression and emotional eating. The study conducted by Kandeger and colleagues (2018) found that night eating symptoms directly affect chronotype differences and insomnia severity and indirectly affect disordered eating attitudes by increasing insomnia scores [19]. Another study found that the chronotype of patients with bipolar disorder had both a direct effect and an indirect effect on their night eating symptoms [43]. The indirect effect was partially mediated by the quality of sleep. Additionally, the study showed that seasonality directly impacts night eating symptoms in the same patients.

To date, no in-depth research has explored the connection between an individual's chronotype, night eating, and depressive symptoms. Understanding the underlying mechanisms of these potential links is crucial to treating and preventing night eating effectively. The current study aims to clarify the relationship between night eating symptoms and chronotype by examining the role of depressive symptoms in a sample of university students. Specifically, we hypothesized that the dimension of eveningness could be related to night eating symptoms through the mediation role of depressive symptoms.

## Methods

### Study design and participants

This research was a cross-sectional study. Participants were recruited among university students from the University of L’Aquila. The data were collected from 905 students at the University of L’Aquila. Participants were invited to take part in this study between early November 2022 and late February 2023. During this period, we contacted 1210 students, of whom 921 agreed to participate after receiving an explanation of the study; 16 students were excluded from the study due to their ages exceeding the predetermined range of 18 to 35 years, which was established to control for age-related variables. No monetary compensation or academic credit was offered for participation. Participants were requested to provide sociodemographic and anthropometric data, including age, sex, height, and weight, and to complete a psychometric assessment comprising the Night Eating Questionnaire (NEQ), the Morningness–Eveningness Questionnaire (MEQ), and the Beck Depression Inventory (BDI), as detailed below.

The study was conducted in person using paper-based materials, and the order of questionnaire administration was standardized across all participants and predetermined before the study began.

### Ethics

This research was conducted in accordance with the Declaration of Helsinki, and approval was obtained from the ethical committee of the University of L’Aquila. All procedures were performed with the entire understanding of the subjects who had read and signed an informed consent form before participating in this research project. All authors declare that no financial support was received for this study.

## Measures

All participants were invited to complete a sociodemographic survey, including health-related information such as weight and height. Moreover, participants completed a battery consisting of three self-report questionnaires: the Night Eating Questionnaire (NEQ), the Morningness–Eveningness Questionnaire (MEQ), and the Beck Depression Inventory (BDI).

### Night eating symptoms

The Italian version of the Night Eating Questionnaire (NEQ) [51] was utilized to explore night eating behaviors. The NEQ includes 15 items assessing mood, sleep disturbances, morning anorexia, food cravings, food intake after the evening meal, nocturnal awakenings with food ingestion, awareness, and feelings of control during eating episodes. The cutoff was set at 25 points, as suggested by the authors [51], to cast a wide net for screening possible cases of NES. Although the NEQ does not originally assess the extent of perceived distress associated with symptoms or the degree of impairment, the authors included item 15 specifically to evaluate the distress resulting from night eating symptoms.

In the current sample, the standardized Cronbach’s α of the NEQ was 0.74.

### Chronotype

The Italian version of the Morningness-Eveningness Questionnaire (MEQ) [52] was employed to assess the chronotype; it consists of 19 items evaluating individual differences in the timing of the sleep–wake cycle, wakefulness, and peak performance times. The subjects, based on their total scores, are categorized into three groups: Morning Type (scores 59–86), Intermediate Type (scores 42–58) and Evening Type (scores 16–41).

In the current sample, the standardized Cronbach’s α of the MEQ was 0.78.

### Depressive symptoms

The Beck Depression Inventory (BDI) [53] is a scale used to evaluate the severity of depressive symptoms. It consists of 21 items, each characterized by four possible answers, scored from 0 to 3. A total score of 16 and above indicates the presence of moderate or severe depression.

In the current sample, the standardized Cronbach’s α of the BDI was 0.81.

## Data analysis

Descriptive statistics were employed to examine the sociodemographic characteristics of the sample group together with frequencies and percentages. The data are presented as mean ± SD (standard deviation) for continuous variables and percent frequency for categorical variables. One-way analysis of variance was used to compare the MEQ, BDI, NEQ, and body mass index (BMI) scores among the chronotypes. To assess the relationship among night eating symptoms (NEQ), chronotype (MEQ), body mass index (BMI), and depressive symptoms (BDI), correlation coefficients and their significance were computed using Pearson (r) correlation analysis. One-tailed *p*-values less than 0.01 were considered statistically significant. A significance threshold of *p* < 0.01 was adopted, as stricter criteria are typically recommended in exploratory studies seeking to identify potential associations or effects [54].

Mediation analyses were conducted using the SPSS Macro PROCESS (version 3.3). Specifically, we aimed to examine the indirect relationship between chronotype differences and night eating symptoms, with a specific emphasis on the potential influence of depressive symptoms. While mediation analyses can suggest causal pathways, the limitations of the atemporal cross-sectional design used in this study prevent us from determining definitive causal relationships; rather, these analyses explore the interrelationships among multiple variables.

All the statistical analyses were performed with IBM SPSS Statistics for Windows, Version 20.0. (IBM Corp, Armonk, NY, USA).

## Results

The sample comprised 905 subjects, including 283 men (31,3%) and 621 women (68,7%), with a mean age of 25.54 years (SD = 10.18), with an age range between 18 and 35, mean height of 168.24 cm (SD = 8.14), mean weight of 64.54 kg (SD = 32.87), and mean BMI of 21.97 kg/m^2^ (SD = 2.8). In the sample, BMI was distributed as follows: 104 (11.6%) subjects were underweight, 665 (73.5%) were of average weight, and 126 (14.1%) were overweight. Table 1 presents the sample psychometric characteristics. The distribution of chronotypes in the cohort was as follows: Morning Type, 14.9%; Intermediate Type, 64.6%; and Evening Type, 20.2% (Table 1).

**Table 1:** Sample psychometric characteristics

Mean SD range	
*BDI scores*	6.43 ± 4.87 0–19
*MEQ scores*	49.05 ± 9.16 21–82
Morning-type	135 (14.9%)
Intermediate	585 (64.6%)
Evening-type	183 (20.2%)
*NEQ scores*	10.95 ± 4.58 1–33
Met criteria	33 (3.6%)
Did not meet criteria	872 (96.4%)

As reported in Table 2, MEQ and BDI scores were inversely correlated; subjects with lower MEQ scores achieved higher BDI rates, indicating a modest relation between evening chronotype and depressive symptoms. In our population, 33 subjects (3.6%) reached the criteria for NES. The findings revealed a significant inverse correlation between NEQ and MEQ scores (Table 2). Participants meeting the criteria for NES achieved lower MEQ scores, indicating an association between NES and the eveningness dimension. Our results also confirmed that individuals with higher NEQ scores tend to have higher BDI scores, showing a significant positive correlation between NES and depressive symptoms.

**Table 2:** Pearson product-moments correlation coefficients

	1	2	3	4
1. Night Eating Questionnaire	1.00			
2. Morningness–Eveningness Questionnaire	− .241^**^	1.00		
3. Beck Depression Inventory	.370^**^	− .127^**^	1.00	
4. Body mass index	− .044	.092^**^	− .093^**^	1.00

^*﻿^*p* < 0.05; **p < 0.01

The results of the ANOVA indicated statistically significant differences between the groups regarding NEQ and BDI scores. Evening types had higher BDI and night eating scores than the other two chronotypes (Table 3).

**Table 3:** Comparison of scale scores between chronotypes

	Chronotype								F(2,902)	*p*	*η*^2^	Post hoc*
	Evening type (A)			Intermediate type (B)			Morning type (C)					
	Mean		SD		Mean	SD	Mean	SD				
Morningness–Eveningness Questionnaire		36,60	3,77		49,58	4,71	63,64	4,61	1401,76	< .001	0,757	A > B > C
Night Eating Questionnaire		12,73	5,06		10,77	4,29	9,24	4,36	24,87	< .001	0,052	A > B > C
Beck Depression Inventory		7,44	5,24		6,44	4,77	5,03	4,45	9,72	< .001	0,021	A > B > C
Body mass index		21,96	2,86		21,81	2,74	22,59	3,05	4,26	< .05	0,009	A = B = C

Post hoc comparisons were carried out by using the LSD multiple group comparison test (*p* < 0.05)

## Mediation analysis

MEQ (*R*^2^ = 0.376, *F* (2,900) = 96.19, *P* > 0001) had a statistically significant negative direct effect on both BDI (*β* = − 0.238, *P* < 0.001) and NEQ (*β* = − 0.371; *P*-value < 0.05) variables.

**Fig. 1:**
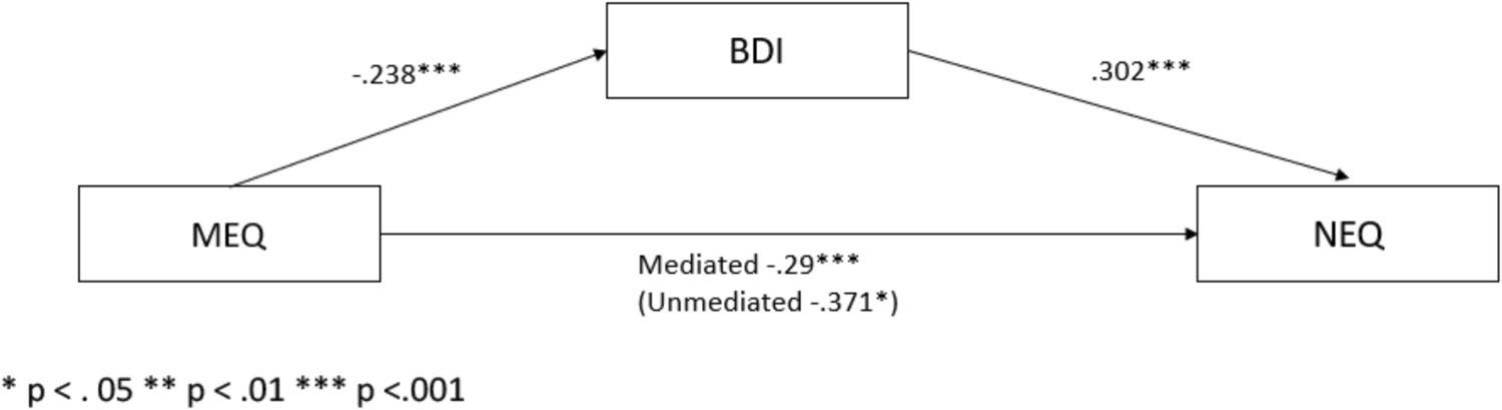
Mediation analysis: standardized regression coefficients for the relationship between evening chronotype assessed by MEQ and night eating behaviors (NEQ) as mediated by depressive symptoms (BDI).

The direct effect of BDI on NEQ was positive and statistically significant (*β* = 0.302; *P* < 0.001). Additionally, in evaluating the mediation effect, the indirect negative effect (− 0.29) of MEQ on NEQ through BDI was determined to be statistically significant (*P* < 0.001). These findings are presented in Fig. 1, which illustrates the mediation analysis.‬‬‬‬

## Discussion

In this study, we evaluated the role of depressive symptoms in the relationship between chronotype and night eating symptoms in a sample of university students.

The general population prevalence reported for NES is 1.5% in the United States [25], 1.1% in Germany [32], 1.5% [49] in Japan, and 0.9% in Australia [26]. Unfortunately, no data regarding the general prevalence among the Italian general population are currently available, representing a gap in the literature.

The prevalence among university students is generally higher compared to the general population [50]. Our results revealed a prevalence rate of 3.6% for NES, which is consistent with the rates reported in other studies in the USA: respectively, 4% [14] and 5.7% [47], and in China 5.4% [48] among groups of college students.

Indeed, prolonged use of computers, tablets, and screens, as observed in academics [55], could lead to circadian lag and increased development of night eating behaviors among college students [56]. Interestingly, a recent study [39] found a higher prevalence of NES (8.1%) in a sample of non-clinical Greek adults, shedding light on the possible impact of latitude, culture, occupation and working hours on the prevalence of NES.

Our study revealed no correlation between night eating symptoms and BMI, suggesting, in line with the literature, that this association may be more evident in clinical samples [9, 20, 57]. Moreover, BMI was correlated with NES assessed with the NEQ in a sample of college students [57]. Furthermore, a previous study [58] proposed that age may mediate the relationship between NES and BMI, indicating a strong association between NES and BMI in subjects aged 55–60, with no correlation in younger subjects. Our results are consistent with this hypothesis.

In line with the current literature, our correlational analysis revealed a significant relationship between night eating symptoms and depressive symptoms [23, 24]. The relationship between depressed mood and night eating behavior has been a topic of debate, but it has been consistently identified in most studies [27, 28, 59]. Numerous studies [36, 60] have found that a high percentage of individuals with NES meet the criteria for major depressive disorder and have suggested that depression, male gender, and a BMI above 25 could be considered risk factors for NES [32]. Although the role of serotonin is still debated in depression [61], this neurotransmitter may be involved in the pathophysiology of night eating. This hypothesis may be supported by single-photon emission computed tomography, which has revealed a significant increase in serotonin transporters in the midbrain of individuals with NES [60]. Increased serotonin transporter levels could contribute to diminished postsynaptic serotonin transmission, potentially impairing circadian rhythms and satiety [62]. These findings may also help explain the strong connection between night eating, depressive symptoms, and circadian rhythm, and may suggest that enhancing serotonin function can effectively treat night eating. [62].

Further, our results provided insights into the relationship between night eating and evening chronotype. In the context of the ongoing literature debate about the role of the eveningness dimension in night eating, we have confirmed the robust relationship between NES and evening chronotype among university students [9, 20]. The current results suggest that individuals with NES might present a circadian delay in food intake and general functioning, highlighting the importance of investigating whether night eating symptoms directly influence chronotype [19]. Researchers have hypothesized that the relationship between night eating and circadian rhythm should be assessed in both directions, considering various factors such as artificial light exposure, melatonin, temperature, and digital utilities that may impact this relationship [12, 63]. Further research is needed to explore this relationship and identify potential mechanisms that underlie it.

Moreover, our study shed light on the critical role of depressive symptoms in the relationship between chronotype and night eating. Specifically, our results in a sample of university students show a pathway from chronotype to night eating, influenced by depressive symptoms. There are several possible explanations for this finding. Night eating symptoms in university students with an evening chronotype may function as a coping strategy for depressive symptoms linked to higher stress levels, as seen in other eating disorders [64]. Otherwise, night eating may be a consequence of depressive symptoms among the many possible manifestations [24].Furthermore, the misalignment of the central and peripheral circadian clocks associated with the evening chronotype, in addition to changes in the HPA axis and increased levels of cortisol, could provide an explanation for these findings [64]. The dysregulation of cortisol peak is a shared characteristic of evening chronotypes [65], depression [66], and NES [67]. This common biological profile could explain the effectiveness of selective serotonin reuptake inhibitors (SSRIs) in treating night eating. SSRIs may work by lowering cortisol levels [68], realigning evening misalignment of the circadian rhythm, and alleviating depressive symptoms, which could contribute to night eating. On the other hand, the mediation of depressive symptoms is partial and not complete. Food addiction has been correlated with the severity of night eating, the dimension of eveningness, and depression [48, 49]. It would be interesting to investigate whether depressive symptoms in university students could mediate the relationship between food addiction and chronotype, also considering the relationship with the severity of night eating [48, 49]. Future studies would be interesting to investigate whether other variables mediate the relationship between night eating symptoms and chronotype, such as food addiction, emotional regulation or insomnia and sleep quality [11, 19, 48, 49]. These findings enrich current knowledge on the impact of depressive symptoms on night eating behaviors [23–25], with important implications for chronotherapeutic approaches such as BLT in treating night eating symptoms [45, 46]. BLT can regulate the circadian rhythm and alleviate symptoms of disorders caused by circadian shifts or disruption while also addressing mood symptoms [69]. Therefore, our results emphasize the importance of increasing the utilization of chronotherapeutic approaches as a cost-effective and well-tolerated therapy for night eating symptoms, as suggested by other researchers [70].

Overall, these results suggest that an increase in depressive symptoms may be a critical factor impacting night eating behaviors in college students with an evening chronotype. Implementing chronotherapeutic interventions like BLT can help protect such students from developing depressive symptoms and night eating. Further research is needed to understand the underlying mechanisms behind this relationship and identify the most effective treatment options for night eating symptoms and NES in different populations.

## Strengths and limitations

Our study has some limitations that need to be considered. Firstly, it was conducted solely on university students, which might make it difficult to generalize the findings to a larger population. Additionally, we did not conduct psychiatric interviews with the participants, and the entire dataset was based on self-reported data, which could have potential biases. However, the validity of self-reported data for medical conditions has been demonstrated in several studies [71, 72], and they are commonly used in screening and surveillance programs [73, 74].

Despite some biases reported in the self-reporting of height and weight, particularly at extreme values, estimated anthropometric measurements used to calculate BMI are generally accepted and considered sufficiently reliable [75]. Further limitations include the sample having a higher prevalence of women, who are particularly vulnerable to nocturnal eating disorders and depression [23]. The restricted list of variables and the non-inclusion of other variables that could have served as mediating or moderating factors constitute another limitation.

In this context, future clinical studies should aim to validate our findings to further clarify the relationship between chronotype differences and night eating symptoms. Furthermore, it is crucial for these studies to investigate additional relevant variables, such as emotional regulation, anxiety, and personality characteristics, to assess their potential impact on night eating behaviors. Lastly, the cross-sectional study design may be considered a limitation. More detailed findings can be obtained from longitudinal and prospective studies.

## What is already known on this subject?

The existing literature regarding chronotypes, NES and depression has been mainly conducted on university students and adolescents [16, 17]. Morning preference has a negative correlation with both depression and emotional eating in the general population [42]. Night eating symptoms have a direct effect on the chronotype differences [13] among university students, and the chronotype of patients with bipolar disorder had both a direct effect and an indirect effect on their night eating symptoms [37].

## What this study adds

This study aims to investigate the relationship between night eating symptoms and chronotypes by examining the role of depressive symptoms in university students. Given its partial mediating role, increased depressive symptoms, together with other variables may possibly explain the relation between evening chronotype and night eating in university students.

## Data Availability

The datasets generated during and/or analysed during the current study are available from the corresponding author upon reasonable request.

## Abbreviations

NEQ: Night Eating Questionnaire
MEQ: Morningness–Eveningness Questionnaire
BDI: Beck Depression Inventory
NES: Night eating syndrome
BLT: Bright light therapy
BMI: Body mass index
SSRIs: Selective serotonin reuptake inhibitors
